# Disruption of the structural and functional features of surfactant protein A by acrolein in cigarette smoke

**DOI:** 10.1038/s41598-017-08588-5

**Published:** 2017-08-16

**Authors:** Rina Takamiya, Koji Uchida, Takahiro Shibata, Toshitaka Maeno, Masaki Kato, Yoshiki Yamaguchi, Shigeru Ariki, Yoshihiro Hasegawa, Atsushi Saito, Soichi Miwa, Hiroki Takahashi, Takaaki Akaike, Yoshio Kuroki, Motoko Takahashi

**Affiliations:** 1 Department of Biochemistry, Sapporo Medical University, School of Medicine, Hokkaido, 060-8556 Japan; 2 Graduate School of Bioagricultural Sciences, Nagoya University, Nagoya, Aichi Japan; 3 Department of Medicine and Biological Science, Gunma University Graduate School of Medicine, Maebashi, Gunma Japan; 4 Structural Glycobiology Team, RIKEN-Max Planck Joint Research Center for Systems Chemical Biology, RIKEN Global Research Cluster, Wako, Saitama Japan; 5 Department of Respiratory Medicine and Allergology, Sapporo Medical University School of Medicine, Sapporo, Hokkaido Japan; 6 Department of Cellular Pharmacology, Graduate School of Medicine, Hokkaido University, Sapporo, Hokkaido Japan; 7 Department of Environmental Health Sciences and Molecular Toxicology, Tohoku University Graduate School of Medicine, Sendai, Japan

## Abstract

The extent to which defective innate immune responses contribute to chronic obstructive pulmonary disease (COPD) is not fully understood. Pulmonary surfactant protein A (SP-A) plays an important role in regulating innate immunity in the lungs. In this study, we hypothesised that cigarette smoke (CS) and its component acrolein might influence pulmonary innate immunity by affecting the function of SP-A. Indeed, acrolein-modified SP-A was detected in the lungs of mice exposed to CS for 1 week. To further confirm this finding, recombinant human SP-A (hSP-A) was incubated with CS extract (CSE) or acrolein and then analysed by western blotting and nanoscale liquid chromatography-matrix-assisted laser desorption/ionisation time-of-flight tandem mass spectrometry. These analyses revealed that CSE and acrolein induced hSP-A oligomerisation and that acrolein induced the modification of six residues in hSP-A: His39, His116, Cys155, Lys180, Lys221, and Cys224. These modifications had significant effects on the innate immune functions of hSP-A. CSE- or acrolein-induced modification of hSP-A significantly decreased hSP-A’s ability to inhibit bacterial growth and to enhance macrophage phagocytosis. These findings suggest that CS-induced structural and functional defects in SP-A contribute to the dysfunctional innate immune responses observed in the lung during cigarette smoking.

## Introduction

Chronic obstructive pulmonary disease (COPD) describes lung disorders characterised by incompletely reversible airflow obstruction. COPD affects populations worldwide^1^ and is predicted by the World Health Organization to become the third leading cause of death by 2030. Cigarette smoke (CS) is a leading cause of COPD. CS contains a complex mixture of more than 5,000 chemicals, including reactive oxygen species (ROS) and carbonyl compounds, which directly injure lung epithelial surfaces^2^. Acrolein is the strongest electrophile among all αβ-unsaturated aldehydes in CS^3^ and is present at concentrations of 1–10 μM in the airway secretions and tracheal aspirates of smokers^4^. Acrolein reacts with nucleophilic sites within proteins, such as cysteine, lysine, and histidine residues, through Michael addition and Schiff base formation^3, 5, 6^. Recent studies have indicated that acrolein modification induces functional defects in its targets, such as extracellular matrix (ECM) protein^7^, apolipoprotein E^8^ and protein disulphide isomerase, during CS exposure^9^. However, the mechanisms by which CS-induced functional changes in proteins are involved in the pathogenesis of COPD, such as the susceptibility to bacterial infection, have not fully been elucidated.

As the primary organs of respiration, the lungs are directly exposed to the air and its constituent pollutants, pathogens, and allergens. The pulmonary surfactant proteins SP-A and SP-D play important roles in innate immune responses in the airway and alveolar space that protect the lungs from exposure to harmful pathogens^10, 11^. SP-A and SP-D belong to the C-type lectin superfamily and have host defence functions, including the regulation of mediator production, the phagocytosis of apoptotic cells, and anti-microbial activities^12^, over the surface area of the lung. SP-A, but not SP-D, binds to carbohydrates and to non-carbohydrates such as dipalmitoylphosphatidylcholine^13, 14^ and lipid A^15^. Thus, SP-A plays an important role in the clearance of several varieties of pulmonary bacteria, such as *S. aureus*, *Pseudomonas aeruginosa*, *Streptococcus* and *Escherichia coli* (*E. coli*) by interacting with such microbial pathogen-associated molecular pattern molecules (PAMPs)^16–18^.

Recent studies have reported that cigarette smoke exposure can alter the SP-A levels. The SP-A content in BAL fluids is significantly lower in smokers than non-smokers^19^. In addition, rats chronically exposed to CS also have decreased levels of SP-A in BAL fluids, but the lung tissue levels of SP-A are not changed^20^. In contrast, some reports have indicated that serum and sputum SP-A levels are significantly higher in smokers than in non-smokers^21–23^. In COPD patients, the SP-A content in BAL fluids is lower in smokers with emphysema than in those without emphysema^24^. In addition, the amount of SP-A in exhaled endogenous particles is lower in patients with COPD compared with healthy non-smokers^25^. However, in another study, the lung tissues levels of SP-A have been found to be higher in COPD patients compared with controls^26^. The expression of SP-A is also significantly elevated in the lung of CS-exposed mice with emphysema compared with air-exposed mice^27^. As described above, although recent studies have suggested that SP-A may be a potential biomarker for COPD, these data reveal a lack of consensus regarding the SP-A levels in smokers and COPD patients and the functional effects of CS on SP-A.

Recent studies have indicated that CS increases the risk of microbial infection by compromising the innate immune response, and this effect is considered to be a major contributor to the pathogenesis of COPD^28, 29^. Moreover, Kuzmenko *et al*.^30^ have indicated that the exposure of SP-A to phospholipid radicals results in oxidative modification and functional inactivation of SP-A. In the present study, we sought to determine whether CS directly modifies the structure of SP-A and if so, whether the modification(s) impairs the innate immune functions of SP-A.

## Results

### Detection of acrolein-modified SP-A in the lungs of CS-exposed mice

**Figure 1:**
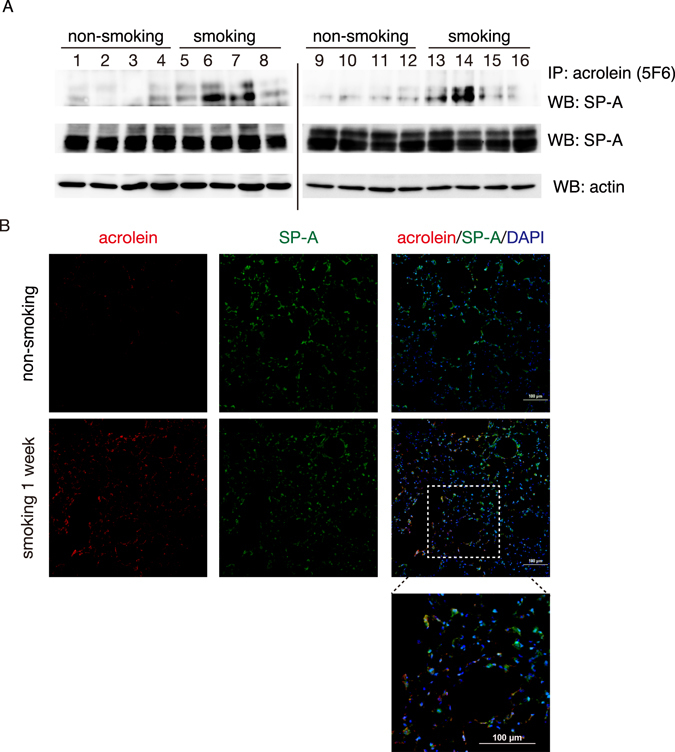
The expression of acrolein-modified SP-A in mouse lungs after 1 week of exposure to CS. Eight-week-old female C57BL/6 mice were exposed to the smoke from four unfiltered cigarettes per day for one week. (**A**) Upper panel: Lung tissue lysates were immunoprecipitated with anti-acrolein antibody (mAb5F6) and subjected to western blotting with anti-SP-A antibody. Lower panel: Lung tissue lysates were subjected to western blot analysis using anti-SP-A and anti-actin antibodies. Lanes 1–16 represent individual samples from n = 8 mice per group. Uncropped blots are shown in Supplementary Fig. S5. (**B**) Immunofluorescence staining of lung sections with anti-acrolein antibody (red), anti-SP-A antibody (green), and DAPI (blue). Scale bars: 100 μm. These experiments were performed with n = 8 mice per group, and representative results are shown.

Acrolein is present at concentrations of 1–10 μM in the airway secretions and tracheal aspirates of smokers^4^, and acrolein adducts are found in bronchoepithelial cells, epithelial and smooth muscle cells of the small vessels and inflammatory cells in the lungs of patients with COPD^31^. Because acrolein is also endogenously formed through oxidation reactions, to determine whether acrolein in CS directly affects SP-A structure and/or function, we examined the lungs of mice exposed to CS for 1 week. In this model, we observed no differences in morphology (data not shown), SP-A levels in the lung (Fig. 1A), or BAL fluid (data not shown) of lungs exposed to CS for 1 week and non-exposed lungs. To determine whether SP-A was modified by acrolein during CS exposure, we immunoprecipitated lung lysates with mAb5F6, an anti-acrolein monoclonal antibody specific for Nε-(3-formyl-3,4-dehydropiperidino)lysine (FDP-lysine), the main product formed by the reaction of acrolein with lysine residues^3, 32^. The immunoprecipitated materials were then subjected to western blot analysis with an anti-SP-A antibody. Acrolein-modified SP-A was more abundant in the lung extracts from CS-exposed mice than non-exposed mice (Fig. 1A). However, several lung lysates in CS-exposed mice showed a decreased abundance of acrolein-modified SP-A. Therefore, we determined the levels of aldehyde adducts of SP-A by using the aldehyde reactive probe *N*-(aminoxyacetyl)-*N’*-biotinylhydrazine (ARP). Similar to the Fig. 1A, ARP-modified SP-A was also induced in CS-exposed lungs compared with non-exposed lung (Supplementary Fig. S1). We next performed immunofluorescence staining of lung sections to examine the localisation of SP-A and acrolein adducts after CS exposure. As shown in Fig. 1B, SP-A was expressed at similar levels in the alveolar cells of CS-exposed lungs and non-exposed lungs, whereas acrolein adducts were detected at markedly higher levels in the bronchioles and alveolar cells of CS-exposed lungs compared with non-exposed lungs. Moreover, acrolein staining partially overlapped SP-A staining in alveolar cells. These results indicated that lung SP-A was modified by acrolein after CS exposure for 1 week.

### Acrolein-hSP-A adducts after treatment with CSE or acrolein

**Figure 2:**
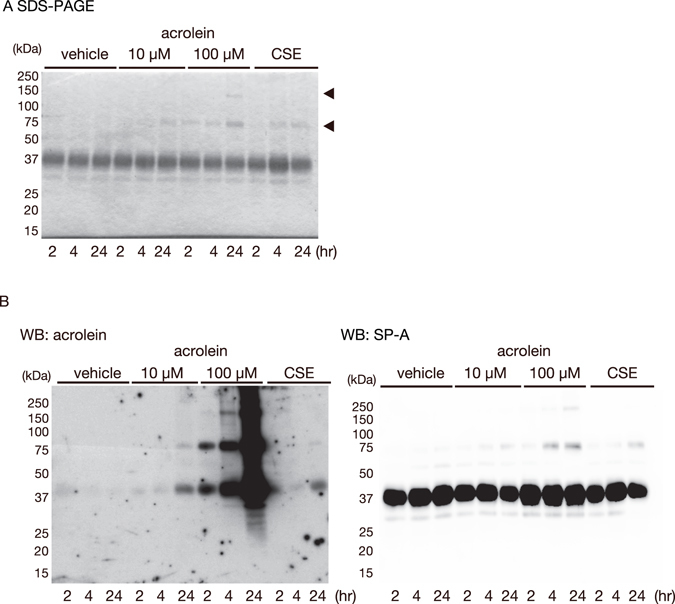
The acrolein modification of recombinant hSP-A by acrolein and CSE. Recombinant human SP-A (5 µM) was incubated with vehicle, acrolein (10 or 100 µM), or CSE (10%) at 37 °C for the indicated times, and 10 µl aliquots were then subjected to SDS-PAGE. (**A**) A gel stained with Coomassie Brilliant Blue. Arrows indicate the positions of the oligomers. (**B**) Western blot analysis using anti-acrolein antibody (left) or anti-SPA antibody (right). These experiments were performed seven times, and representative results are shown.

To clarify whether CS and acrolein could directly modify SP-A, we incubated purified recombinant human SP-A (hSP-A, 5 μM) with vehicle, acrolein (10 or 100 μM; equivalent to a 2:1 or 20:1 molar ratio of acrolein to SP-A, respectively), or CS extract (10%) at 37 °C for 2, 4, or 24 h. Of note, a recent study has shown that buffers containing >100 mM Tris-HCl and/or with pH >8.0 interfere with the formation of acrolein–bovine serum albumin adducts^33^. In this study, we performed these incubations in 5 mM Tris-HCl buffer at pH 7.4. At the end of the incubation, the treated hSP-A samples were subjected to reducing SDS-PAGE and western blotting with anti-SP-A antibody. We detected no significant differences in the formation of acrolein–hSP-A adducts when the incubation was performed in phosphate-buffered saline and 5 mM Tris-HCl buffer, pH 7.4 (data not shown). As shown in Fig. 2A, the staining of SDS-PAGE gels revealed that the major species of hSP-A was monomeric, regardless of the presence of acrolein or CSE. Although hSP-A dimers were formed after incubation with CSE or 10 μM acrolein for 24 h, several oligomeric forms were detectable after incubation with 100 μM acrolein for 24 h (Fig. 2A, arrows). Next, to determine whether lysine residues in hSP-A were modified by acrolein, we performed western blotting with anti-acrolein antibody (mAb5F6). Acrolein adducts of the hSP-A monomer and dimer were detected in the 10 μM acrolein- and CSE- treated samples, and several oligomers were detectable after incubation with 100 μM acrolein for 24 h (Fig. 2B left). These data suggested that exposure to CSE induces acrolein modification of hSP-A.

### Aldehyde-hSP-A adducts after treatment with CSE or acrolein using an aldehyde reactive probe (ARP)

**Figure 3:**
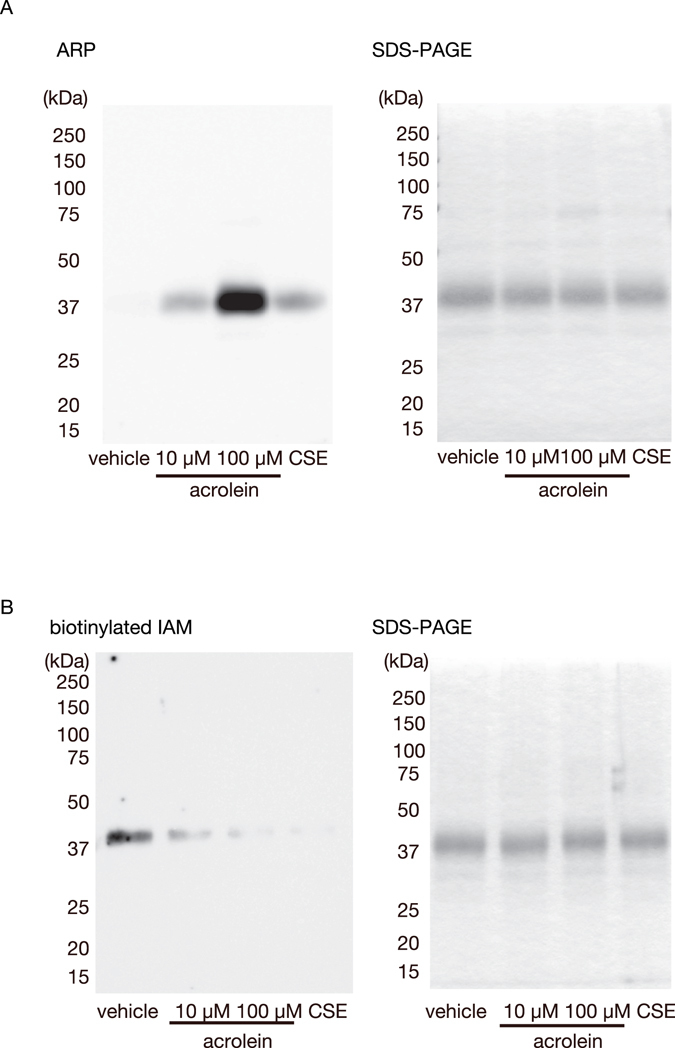
The modification of recombinant hSP-A by acrolein and CSE. Recombinant human SP-A (5 µM) was incubated with vehicle, acrolein (10 or 100 μM), or CSE (10%) at 37 °C for 4 h. (**A**) The amount of aldehyde-protein adducts was determined by using ARP. ARP-labeled SP-A was detected by streptavidin blotting (left). A gel stained with Coomassie Brilliant Blue (right). (**B**) The level of the oxidation of thiols in SP-A was determined by using IAM. IAM-labeled SP-A was detected by streptavidin blotting (left). A gel stained with Coomassie Brilliant Blue (right). These experiments were performed three times each, and representative results are shown.

As the anti-acrolein monoclonal antibody mAb5F6 specifically reacts with FDP-lysine, we next determined whether acrolein and CSE induced aldehyde adducts in SP-A by using an ARP. As in Fig. 2, ARP-labeled hSP-A was markedly induced after a 4 h- incubation with acrolein (10 or 100 μM) or CSE (Fig. 3A left). Thus, acrolein and CSE increased the levels of aldehyde-hSP-A adducts.

### Free thiols in hSP-A after treatment with acrolein or CSE

Cysteine thiols are well known targets of acrolein. To determine whether acrolein and CSE modify the free thiols in SP-A, we first incubated hSP-A with vehicle, acrolein, or CSE and then added biotinylated iodoacetamide (IAM), which binds specifically to reactive thiols^34^. Notably, the biotinylated IAM labelling of hSP-A was markedly decreased after a 4 h incubation with acrolein (10 or 100 μM) or CSE (Fig. 3B left). Thus, CSE and acrolein modified the free thiols in hSP-A.

### Secondary structure of acrolein-modified hSP-A

A recent study has indicated that CS and acrolein induce structural changes in the protein disulphide isomerase by decreasing its ellipticity and helical content^9^. To determine whether acrolein alters the structure of hSP-A, we performed circular dichroism (CD), a technique that is based on the absorption of polarised light and is commonly used to determine the secondary structure and folding properties of proteins^35, 36^. Although acrolein adducts were detected by western blotting after 4 h incubating acrolein with and hSP-A (20:1 molar ratio of acrolein to SP-A) (Fig. 2C), there were no significant differences in the CD spectra of hSP-A incubated with vehicle or acrolein for the same time (Supplementary Fig. S2). These results therefore suggested that acrolein modification does not affect the secondary structure of hSP-A.

### Identification of acrolein modification sites in hSP-A

**Figure 4:**
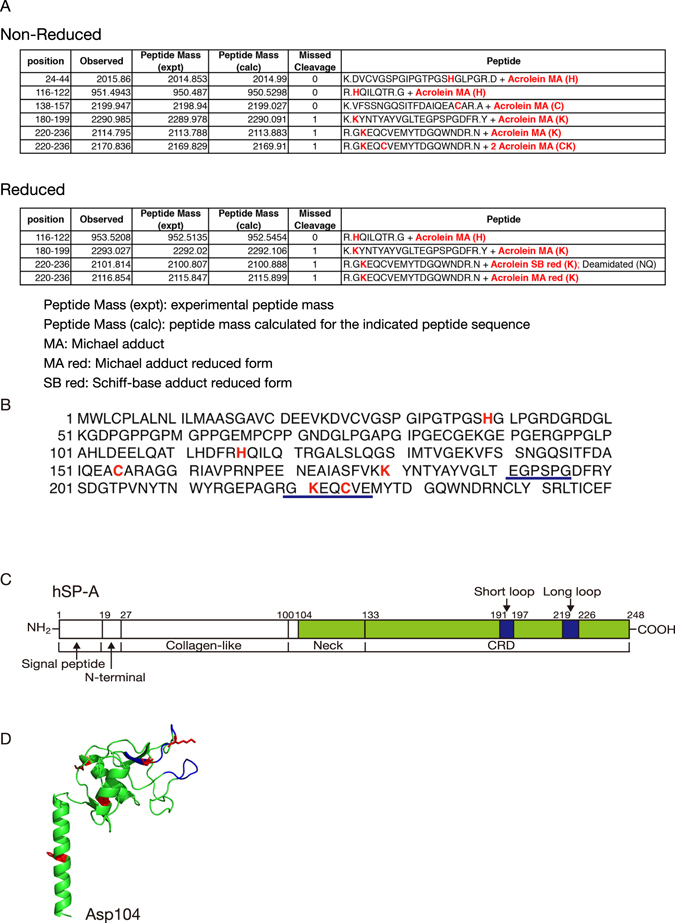
The identification of acrolein-modified residues in SP-A. (**A**) Nano-LC/MALDI-TOF MS/MS analysis of peptides obtained from the trypsin digestion of acrolein-modified hSP-A. Trypsin digested acrolein-modified SP-A was subjected to Nano-LC/MALDI-TOF MS/MS analysis and identified by using MASCOT Daemon. Acrolein-modified residues are shown in red. (Upper) The MS/MS analysis of non-reduced acrolein-modified SP-A. (Lower) The MS/MS analysis of NaBH_4-_reduced acrolein-modified SP-A. (**B**) The amino acid sequence of hSP-A. Acrolein-modified residues are shown in red, and the double-loop structure is indicated by the blue underlined text. (**C**) The structural organisation of human SP-A. SP-A is conceptually divided into a signal peptide, N-terminal, collagen-like neck and CRD domain. The double-loop structure in the CRD domain is shown in blue (long loop residues: 219–226 and short loop residues: 191–197). Green corresponds to residues 104–248, which are shown in the crystal structure in (**D**). (**D**) A homology model of the hSP-A neck and CRD domains (residues: 104–248) based on the crystal structure of rat SP-A (PDB ID: 1R13). The model was constructed using SWISS-MODEL^37^. The protein backbone is shown in green, the double-loop structure in the CRD domain is shown in blue, and acrolein-modified residues are highlighted in red.

The potential targets of acrolein within proteins include the nucleophilic side chains of cysteine, histidine, and lysine residues. To identify the acrolein modification sites in hSP-A, we used nano-LC/MALDI-TOF MS/MS and analysed the MS/MS data using the MASCOT Daemon software. hSP-A was treated with acrolein (20:1 molar ratio of acrolein to SP-A) at 37 °C for 4 h, incubated with or without NaBH_4_, and then digested with trypsin to generate peptides. The MALDI-TOF MS/MS analysis of trypsin digested acrolein-modified hSP-A was carried out in comparison with unmodified SP-A (Supplementary Fig. S4). Under non-reducing conditions (Fig. 4A (Upper) and Supplementary Fig. S4A), there were five Michael addition-type acrolein-modified residues, histidine (His39), cysteine (Cys155 and Cys244), and Lysine (Lys180 and Lysine221), that formed M + 56 Da adducts. Under reducing conditions (Fig. 4A (Lower) and Supplementary Figs. S3 and S4B), there were three Michael addition-type acrolein-modified residues, histidine (His116) and lysine (Lys180 and Lys221), that formed M + 58 Da adducts and one Schiff base reaction-type acrolein modified lysine residue (Lys221) that formed a M + 43 Da adduct. Overall, these results demonstrated that acrolein induced the modification of six residues (His39, His116, Cys155, Lys180, Lys221, and Cys224) in hSP-A (Fig. 4B). As shown in Fig. 4D, a homology model of the hSP-A neck and CRD domains (residues 104–248) based on the crystal structure of rat SP-A (PDB ID 1R13) was constructed by using SWISS-MODEL^37^. The CRD in SP-A has a double-loop structure containing short and long loops (residues 191–197 and 219–226, respectively). Two of the acrolein-modified residues (K221 and C224) are located in the long loop.

### Effects of CSE and acrolein modification on the anti-microbial functions of SP-A

**Figure 5:**
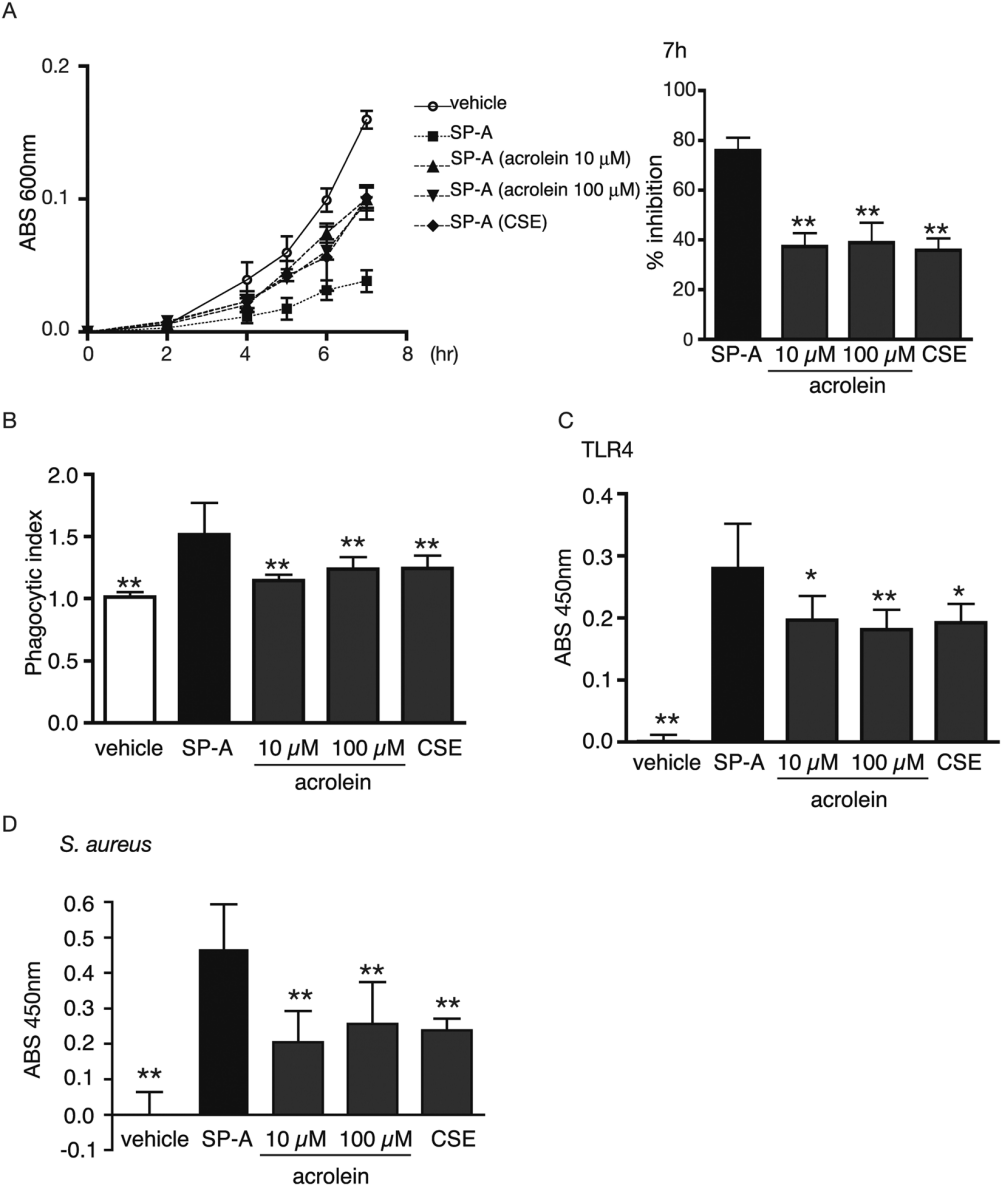
The CSE or acrolein modification of SP-A reduces SP-A–induced inhibition of *E. coli* growth and phagocytosis by RAW264.7 cells. Recombinant human SP-A (5 µM) was incubated with vehicle, acrolein (10 or 100 µM), or CSE (10%) at 37 °C for 4 h and then dialyzed to remove the excess unreacted CSE or acrolein. (**A**) Cultures of *E. coli* were mixed with vehicle or 10 µg/ml of unmodified SP-A, acrolein (10 or 100 µM)-modified SP-A, or CSE-modified SP-A and incubated at 37 °C for 7 h. *E. coli* growth was measured by absorbance at 600 nm (left). The growth inhibition rate of *E. coli* in non-modified SP-A at 7 h was referred to as 100% (right). n = 3 per group. **P < 0.01 vs. unmodified SP-A (n = 3). (**B**) RAW264.7 cells were treated with vehicle or 50 µg/ml of unmodified, acrolein (10 or 100 μM)-modified or CSE-modified SP-A for 2 h. The supernatant was removed, and the cells were incubated with fluorescein-labeled *E. coli* particles for 2 h. The phagocytic index is a measure of the fluorescence intensity of experimental samples relative to the fluorescence intensity of vehicle-treated cells. **P < 0.01 vs. unmodified SP-A (n = 3). (**C**) The binding ability of SP-A to hTLR4. hTLR4 was used to coat microplates, and 5 μg of unmodified, acrolein (10 or 100 μM)-modified, or CSE-modified SP-A was incubated in the plates for 5 h. The binding affinity of SP-A for *S. aureus* was determined by using anti-SP-A polyclonal antibody. The background absorbance (vehicle treated-hTLR4) was subtracted. *P < 0.05, **P < 0.01 vs. unmodified SP-A (n = 3). (**D**) The binding ability of SP-A to *S. aureus*. UV-killed *S. aureus* was coated onto microplates and incubated with vehicle or 2.5 µg of unmodified, acrolein (10 or 100 μM)-modified, or CSE-modified SP-A for 1 h. The binding ability of SP-A to *S. aureus* was assessed with anti-SP-A polyclonal antibody. The background absorbance (vehicle treated-*S. aureus*) was subtracted. **P < 0.01 vs. unmodified SP-A (n = 4). All values shown are the mean ± SD. Statistical significance was determined by using a non-parametric two-way ANOVA followed by Bonferroni’s multiple comparison test.

We next determined whether modification by CSE and acrolein impaired the innate immune roles of SP-A. To assess the role of SP-A, we used 4 h treatments with acrolein (10 or 100 μM) or CSE with SP-A. Subsequently, dialysis was used to remove unreacted acrolein or CSE. The typical roles of SP-A in innate immunity are to inhibit bacterial proliferation^38^ and to enhance the phagocytic activity of macrophages^39^. As first, we investigated the effects of the hSP-A modifications on its ability to inhibit bacterial growth. Unmodified or modified hSP-A was incubated with *E. coli*, and bacterial growth was monitored for up to 7 h (Fig. 5A, left). As shown in Fig. 5A right, the co-incubation of *E. coli* with unmodified hSP-A (10 μg/ml) inhibited bacterial growth by 76.0 ± 3.0% at 7 h. However, acrolein (10 or 100 μM)-modified and CSE-modified SP-A decreased the inhibitory effect of hSP-A by 37.4 ± 3.1%, 38.9 ± 4.7%, and 35.9 ± 2.7%, respectively (Fig. 5A right). Next, we examined the effects of hSP-A acrolein modification on its pro-phagocytic function by using the mouse macrophage cell line RAW264.7. 2 h of treatment with unmodified-, acrolein (10 or 100 μM)-modified, or CSE-modified SP-A had no effect on the cell viability of RAW264.7 cells (data not shown). As shown in Fig. 5B, the pretreatment of cells with 50 μg/ml unmodified hSP-A significantly increased the phagocytic index by 1.51 ± 0.15-fold, as compared with vehicle-treated cells. However, acrolein (10 or 100 μM)- or CSE-modified SP-A significantly decreased the ability of SP-A to enhance the phagocytic activity of RAW264.7 cells (1.14 ± 0.03-fold, 1.24 ± 0.06-fold, and 1.24 ± 0.06-fold, respectively; Fig. 5B). It is well established that SP-A directly binds to several pathogens and receptors^12^. Thus, we next examined the binding abilities of SP-A to TLR4 and *S. aureus* after incubation with acrolein or CSE. Because SP-A directly interacts with TLR4 and modulates phagocytosis through the TLR4-mediated deleterious inflammatory response^40, 41^, we examined the binding of SP-A to TLR4. As shown Fig. 5C, pre-incubation of hSP-A with 10 µM acrolein, 100 µM acrolein, and CSE decreased the binding ability of hSP-A to TLR4 by 77.1 ± 4.6%, 72.6 ± 5.2%, and 71.9 ± 2.5%, respectively. *S. aureus* is a common bacterium in patients with influenza pneumonia^42^, and a recent study has reported that SP-A binds to *S. aureus* and induces its opsonisation^43^. We next assessed the binding ability of SP-A to *S. aureus*. Pre-incubation of hSP-A with 10 µM acrolein, 100 µM acrolein, and CSE decreased the binding ability of hSP-A to *S. aureus* by 75.4 ± 3.5%, 77.7 ± 5.3%, and 71.9 ± 5.1%, respectively (Fig. 5D). These data indicated that the innate immune activities of SP-A were markedly attenuated by exposure to CSE or acrolein.

## Discussion

CS has been shown to cause defects in the function of the innate immunity in the lung^28, 44–46^, but how it promotes infection and lung disease is not fully understood. The results of this study suggest that the modification of SP-A by acrolein is one mechanism by which CS may be detrimental to the innate immune response. Acrolein present in CS is one of the most important environmental risk factors for COPD^47^; thus, we examined whether acrolein directly affected SP-A. We found that acrolein- and aldehyde-modified SP-A were detectable in the alveolar cells in the mouse lung within 1 week of CS exposure (Fig. 1 and Supplementary Fig. S1).

**Figure 6:**
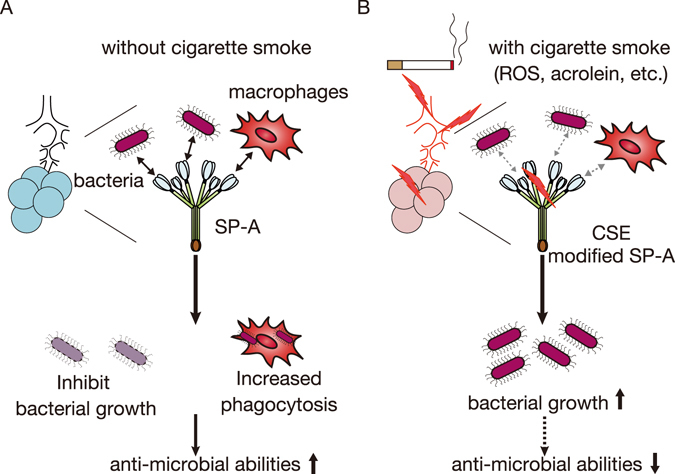
A model of the possible involvement of damaged SP-A in CS-induced innate immune dysfunction. (**A**) The lung is continuously exposed to airborne pathogens. SP-A protects the lung by inhibiting bacterial growth and inducing macrophage phagocytic activity^12^. (**B**) SP-A damage by CS attenuates its ability to inhibit bacterial growth and induce macrophage phagocytosis. The structural and functional disruption of SP-A may thus contribute to CS-induced innate immune dysfunction.

Fully assembled SP-A is an octadecamer that forms a bouquet-like structure (Fig. 6). SP-A has a collagen-like N-terminal region and a C-type carbohydrate recognition domain (CRD) (Fig. 4C). We and others have suggested that supratrimeric oligomerisation may be important for the function of SP-A in innate immunity^40, 48^. In the present study, we demonstrated that incubation with CSE or acrolein induces the formation of high molecular weight oligomers, acrolein–lysine adducts, and aldehyde adducts in hSP-A (Figs 2 and 3). However, acrolein modification did not affect the secondary structure of hSP-A, as shown by CD. We assessed the site(s) of acrolein modification by using nano-LC/MALDI-TOF MS/MS analysis and identified six specific acrolein-modified sites (H39, H116, C155, K180, K221, and C224). The CRD in SP-A has a double-loop structure containing short and long loops (residues 191–197 and 219–226, respectively) that are required for Ca^2+^-dependent carbohydrate binding^17, 49^. Two of the acrolein-modified residues (K221 and C224) are located in the long loop (Fig. 4D), thus raising the possibility that CS exposure might alter carbohydrate binding.

Several studies have investigated the mechanisms by which CS increases the risk of microbial infection^28, 50^. Recent reports have shown that COPD exacerbation is associated with increased systemic inflammation triggered by respiratory bacteria and viruses^51, 52^. In addition, Kirkham *et al*.^7^ have indicated that CS-, acrolein-, or 4-hydroxynoneral-modified ECM proteins promote macrophage adhesion and result in impaired to phagocytosis of macrophage^7^. SP-A also plays an important role in innate immune responses to eliminate microbes in the lung^12^, and consequently, SP-A–deficient mice exhibit defects in microbial clearance^11^. Among the roles played by SP-A in the innate immune response, we focused on its bacterial growth-inhibitory and macrophage pro-phagocytic activities. Notably, a 4 h incubation with CSE or acrolein was sufficient to significantly decrease both activities of hSP-A (Fig. 5A and B). Furthermore, the binding affinities of SP-A to TLR4 and *S. aureus* were also significantly decreased (Fig. 5C and D). Because we found that acrolein modification significantly impaired the innate immune functions of hSP-A, our results suggested that the acrolein modification of the CRD domain might be involved in the decreased ability of modified SP-A to bind to microbes and receptors and promote macrophage phagocytosis.

Under normal circumstances, SP-A has anti-microbial abilities, such as inhibiting bacterial growth and enhancing macrophage phagocytosis in the lung^12^ (Fig. 6A). However, during CS exposure, lungs are exposed to various type of chemicals, including ROS and acrolein^2^. We showed that CS increases the acrolein modification of SP-A, especially in the long loop, thereby impairing the innate immune abilities of SP-A (Fig. 6B). Because innate immune responses are key regulators of the pathogenesis of COPD, our observations suggest that acrolein-modified SP-A may be involved in CS-mediated suppression of innate immunity. Further investigation will be required to determine whether the structural and functional defects of SP-A induced by CS are involved in the pathogenesis of COPD.

## Experimental Procedures

### Mouse exposure to CS

All protocols for animal experiments on mice were approved by the Ethics Committee on Animal Research at Gunma University School of Medicine. All animal experiments were performed according to the guidelines of the Ethics Committee on Animal Research at Gunma University School of Medicine. Female C57BL/6 mice were purchased from Japan SLC, Inc. (Shizuoka, Japan). Mice were exposed to CS (research-grade 3RF4 cigarettes were purchased from Kentucky Tobacco Research and Development Center, University of Kentucky, Lexington, KY), by using a smoking machine designed by Dr. Steven D. Shapiro *et al*. (St. Louis, MO) as described previously^53^. Briefly, eight-week-old female C57BL/6 mice were exposed to the smoke from 4 unfiltered cigarettes for one hour everyday over a period of a week. The control mice were not exposed to air in the chamber, but maintained with the CS-exposed mice. The mice tolerated CS exposure without any obvious evidence of toxicity (carboxyhaemoglobin levels of 10% and no weight loss).

### Western blot analysis

Mice were sacrificed 24 h after the final CS exposure to remove acute inflammation caused by smoke. Lung tissues were collected and homogenized in a lysis buffer (Cell lysis buffer: 20 mM Tris-HCl (pH 7.5), 150 mM NaCl, 1 mM Na_2_EDTA, 1 mM EGTA, 1% Triton, 2.5 mM sodium pyrophosphate, 1 mM, β-glycerophosphate, 1 mM Na_3_VO_4_, and 1 µg/ml leupeptin; obtained from Cell Signaling Technology, Danvers, MA) containing complete protease inhibitor (Roche Diagnostics, Mannheim, Germany). Protein samples were separated by SDS-PAGE and transferred to polyvinylidene difluoride (PVDF) membranes (Bio-Rad Laboratories, Hercules, USA). Then, the membranes were incubated with blocking buffer (Block Ace, DS Pharma Biomedical, Osaka, Japan). Next, the PVDF membranes were probed with the primary antibodies against SP-A (LS-C312616, LifeSpan BioSciences, Inc, Seattle, WA) or β-actin (4967, Cell Signaling Technology). After incubation with a horseradish peroxidase (HRP)-conjugated anti-mouse (W4021) or anti-rabbit (W4011) antibody (Promega, Fitchburg, WI), the immunoreactive bands were visualised using an enhanced chemiluminescence kit (BD Biosciences, Franklin Lakes, NJ) and imaged with an ImageQuant LAS-4000 system (GE Healthcare, Chicago, IL). The primary and secondary antibodies were diluted in immunoreaction enhancer solution (Can Get Signal, Toyobo Life Science, Osaka, Japan).

### Immunoprecipitation

Protein G-Sepharose 4 Fast Flow beads (GE Healthcare) were incubated with an anti-acrolein antibody (mAb5F6; JaICA, Shizuoka, Japan) in washing buffer (Dulbecco’s phosphate-buffered saline containing 0.1% Tween 20) at 4 °C for 1 h and then washed to remove the unbound antibody. Aliquots of lung lysates (500 μg protein) were incubated with anti-acrolein–protein G-Sepharose beads overnight at 4 °C. The beads were washed 5 times with washing buffer and eluted by boiling in SDS sample buffer. Then, the immunoprecipitated materials were separated by SDS-PAGE and subjected to western blot analysis with anti-SP-A antibody as described above.

### Immunofluorescence microscopy

Lung tissues were fixed with 4% paraformaldehyde and sectioned into 5-μm-thick slices. Sections were blocked with 5% BSA and incubated with anti-mouse SP-A and anti-acrolein monoclonal antibodies as described above. Then, the sections were washed and incubated with secondary Alexa 488-conjugated anti-rabbit IgG or Alexa 568-conjugated anti-mouse IgG (Thermo Fisher Scientific, Waltham, MA). The primary and secondary antibodies were diluted in immunoreaction enhancer solution (Can Get Signal immunostain, Toyobo Life Science). The cell nuclei were labeled with 4′,6-diamidino-2-phenylindole (DAPI; Vector Laboratories). Sections were analysed by confocal microscopy (Nikon A1, Nikon, Tokyo, Japan).

### Purification of recombinant hSP-A produced from CHO-K1 cells

The hSP-A purification procedures have been described previously^40^. Briefly, a pEE14 vector containing human SP-A1 cDNA was transfected into CHO-K1 cells, and recombinant hSP-A was expressed using the glutamine synthetase gene expression system. For protein purification, hSP-A–expressing CHO-K1 cells were cultured in serum-free EX-CELL 302 medium (SAFC Biosciences Inc., Buchs, Switzerland) for 3 days. Then, the supernatant was collected and applied to a mannose-Sepharose column in 20 mM Tris-HCl buffer (pH 7.4). The bound proteins were eluted with 5 mM Tris-HCl buffer (pH 7.4) containing 50 mM EDTA and dialyzed against 5 mM Tris-HCl buffer (pH 7.4).

### Preparation of CSE

CSE was prepared as described previously^54^ from research-grade 3RF4 cigarettes. CSE was prepared fresh for each experiment by bubbling the smoke from one cigarette through 10 ml of 5 mM Tris-HCl buffer (pH 7.4) in a 15-ml conical tube for 2.5 min. The CSE solution was filtered through a 0.22-μm pore filter to remove any residual particles. The CSE preparation was measured on the basis of the absorbance at a wavelength of 320 nm. 100% CSE within absorbance values of 2.5 ± 0.2 (320 nm) was used.

### Acrolein and CSE modification of hSP-A

Purified hSP-A (5 μM) was incubated with 10 or 100 μM acrolein or 10% CSE in 5 mM Tris-HCl buffer (pH 7.4) at 37 °C. This buffer was used because buffer containing >100 mM Tris-HCl or at pH ≥8.0 has been shown to interfere with acrolein adduct formation^33^. For use in the assays of bacterial growth, macrophage phagocytosis, and binding to bacteria and TLR4, samples were dialyzed against five changes of the Tris-HCl buffer for 48 h by using a dialysis cassettes (Slide-A-Lyzer Dialysis Cassette, 3,500 MWCO, Thermo Fisher Scientific), to remove the excess unreacted CSE or acrolein.

### Detection of aldehyde-protein adducts by using an aldehyde reactive probe

Aldehyde-protein adducts were detected by labelling with *N*-(aminoxyacetyl)-*N*′-biotinylhydrazine (ARP, Dojindo, Kumamoto, Japan). An aliquot of SP-A (45 μl) was incubated with 5 μl of biotinylated ARP (1 mM) at 37 °C for 1 h. Samples were resolved by SDS-PAGE and transferred to a PVDF membrane, and the membrane was incubated with HRP-conjugated streptavidin. The reactive bands were visualised using an enhanced chemiluminescence kit (BD Biosciences) and analysed using an ImageQuant LAS 4000 system.

### Free thiol labelling with biotin iodoacetamide

Free thiols were detected by labelling with *N*-(biotinoyl)–*N′*-(iodoacetyl) ethylenediamine (Biotin IAM; Molecular Probes)^34^. An aliquot of SP-A (5 μM, 10 μl) was incubated with 40 μl biotinylated IAM labelling buffer (50 mM PIPES (pH 7.4), 50 μM diethylenetriaminepentaacetic acid, and 100 μM biotin IAM) for 30 min at room temperature. β-Mercaptoethanol (50 mM) was then added for 15 min to stop the thiol labelling. The samples were resolved by SDS-PAGE and transferred to a PVDF membrane, and the membrane was incubated with HRP-conjugated streptavidin (BD Biosciences). The reactive bands were visualised using an enhanced chemiluminescence kit (BD Biosciences) and analysed using an ImageQuant LAS 4000 system.

### Nanoscale liquid chromatography–matrix-assisted laser desorption/ionisation time-of-flight tandem mass spectrometry (nano-LC/MALDI-TOF MS/MS) analysis of acrolein adducts

The procedure used for this analysis has been described previously^55^. hSP-A (25 μM) was incubated with acrolein (500 μM) at 37 °C for 4 h. After removal of the excess unreacted acrolein with a PD SpinTrap G25, the samples were reduced with 100 mM NaBH_4_ for 3 h at room temperature, neutralised with 2 N HCl, and precipitated with chloroform/methanol. The precipitated protein was dissolved in 8 M urea and 50 mM NH_4_HCO_3_, reduced with 10 mM Tris(2-carboxyethyl)phosphine hydrochloride, and alkylated with 2-iodoacetamide. The alkylated sample was digested by using sequencing-grade modified trypsin (Promega) in the presence of 0.01% ProteaseMAX surfactant (Promega) for 1 h at 50 °C. The recovered peptides were then resolved by reverse-phase nano-LC (DiNa Nano LC system; KYA Tech, Tokyo, Japan) and then directly fractionated onto a MALDI target plate with α-cyano-4-hydroxycinnamic acid by a spotter (DiNa Nano LC system). MALDI-MS and MS/MS were performed on an ABSCIEX TOF/TOF 5800 system (AB Sciex, Tokyo Japan). The MS/MS data were processed and subjected to database searches by using MASCOT Daemon (Matrixscience).

### Assay of bacterial growth

*E. coli* strain K12 (DH5α; Takara, Shiga, Japan) was transfected with plasmid pcDNA3.1 (Invitrogen) to confer ampicillin resistance^38^ and grown in LB buffer with 50 μg/ml ampicillin. Bacterial growth was monitored by measurement of the absorbance at 600 nm (OD600). When the cultures reached the mid-logarithmic phase (~0.6–0.8 OD), a 10-μl aliquot was added to 2 ml of 10 mM sodium phosphate buffer (pH 7.4) containing 10% LB, 50 μg ampicillin, and 10 μg/ml hSP-A. The tubes were shaken at 300 rpm for up to 7 h, and the OD600 was monitored using a spectrophotometer (U-3010, Hitachi, Tokyo, Japan).

### Macrophage culture

RAW264.7 (a mouse leukaemic monocyte-macrophage cell line, #TIB-71) was obtained from the ATCC (Manassas, VA) and maintained in DMEM supplemented with 10% foetal bovine serum, 100 U/ml penicillin, and 100 μg/ml streptomycin at 37 °C in a humidified atmosphere of 5% CO_2_ in air.

### Macrophage phagocytosis assay

The phagocytic function of RAW264.7 cells was assessed using a Vybrant Phagocytosis Assay Kit (Thermo Fisher Scientific)^54^. RAW264.7 cells were seeded at 5 × 10^4^ cells/well in 96-well microplates in DMEM supplemented with 10% FBS. After 12 h, the medium was exchanged to Macrophage Serum-Free Medium (Thermo Fisher Scientific), and vehicle or modified SP-A (50 μg/ml) was added to the cells for 2 h. The culture medium was removed, and 100 μl of fluorescein-labeled *E. coli* bioparticles (5 mg/ml) was added to the cells for 2 h. After the culture medium was removed, trypan blue solution was added to quench the extracellular probe. The fluorescence intensity of each well was then determined with a fluorescence plate reader using 480 nm as the excitation wavelength and 520 nm as the emission wavelength. The phagocytic index was calculated as the fluorescence intensity of the experimental cells relative to the intensity of vehicle-treated cells.

### Detection of SP-A binding to hTLR4

The binding assay procedures have been described previously^40^. 500 ng recombinant human TLR4 (hTLR4) (Sino Biological Inc., Beijing, China) was coated onto 96-well immune plates (Thermo Fisher scientific), and nonspecific binding was blocked with 10 mM HEPES buffer (pH 7.4) containing 0.15 M NaCl, 5 mM CaCl_2_, and 2% fatty acid-free BSA (buffer A). 5 μg SP-A in buffer A were added and incubated at 37 °C for 5 h. The wells were washed and then incubated with anti-human SP-A polyclonal antibody followed by HRP-labeled anti-rabbit IgG in PBS containing 2% fatty acid-free BSA. The binding of hSP-A to the plates was detected using a TMB Substrate Reagent Set (BD Biosciences). The absorbance intensity of each well was measured at 450 nm.

### Detection of SP-A binding to *S. aureus*

The binding of SP-A to *S. aureus* was assessed by using an ELISA^56^. *S. aureus* (25923, ATCC) was cultured in BactoTM tryptic soy broth (Thermo Fisher Scientific) and killed by UV-irradiation for 10 min. The bacteria were then washed, suspended in PBS, and then stored at −80 °C. The suspension of UV-killed bacteria was allowed to adhere at 1.0 × 10^6^ CFU/well in 96-well microplates overnight and dried. After being coated, the plates were washed with PBS containing 0.1% Triton X-100 and blocked with 5 mM Tris-HCl (pH 7.4) containing 0.15 M NaCl, 2 mM CaCl2, and 2% fatty acid-free BSA (Wako Pure Chemical Industries, Osaka, Japan) (buffer B) for 1 h at 37 °C. 2.5 μg SP-A in buffer B was added and incubated at 37 °C for 1 h. The wells were washed and then incubated with anti-human SP-A polyclonal antibody followed by HRP-labeled anti-rabbit IgG in PBS containing 0.1% Triton X-100 and 2% fatty acid-free BSA. All incubation steps were carried out for 1 h at 37 °C. The binding of hSP-A to the plates was detected by using a TMB Substrate Reagent Set (BD biosciences). The absorbance intensity of each well was measured at 450 nm.

### Statistical analysis

For comparisons between more than two groups and multiple comparisons, an ANOVA test and subsequent Bonferroni’s multiple comparison test were used. Statistical significance was measured using non-parametric testing. The data were analysed using Graphpad Prism 4 (GraphPad Software). Statistical significance was accepted at P < 0.05. The number of replicate samples per group (n) is specified in the figure legends.

## Electronic supplementary material

Supplementary Dataset
