# Supplementary material for: Disruption of the structural and functional features of surfactant protein A by acrolein in cigarette smoke
Source: Sci Rep. 2017 Aug 16;7:8304. doi: 10.1038/s41598-017-08588-5 (PMC5559459; doi:10.1038/s41598-017-08588-5)
Supplement: Supplementary file 1 — Supplementary Dataset [file 41598_2017_8588_MOESM1_ESM.doc]

Supplementary Information

Disruption of the structural and functional features of surfactant protein A by acrolein in cigarette smoke

Rina Takamiya^1*^, Koji Uchida^2^, Takahiro Shibata^2^, Toshitaka Maeno^3^, Masaki Kato^4^, Yoshiki Yamaguchi^4^, Shigeru Ariki^1^, Yoshihiro Hasegawa^1,5^, Atsushi Saito^1,5^, Soichi Miwa^6^, Hiroki Takahashi^5^, Takaaki Akaike^7^, Yoshio Kuroki^1^ & Motoko Takahashi ^1^

^1^Department of Biochemistry, Sapporo Medical University, School of Medicine, Hokkaido, 060-8556, Japan

^2^Graduate School of Bioagricultural Sciences, Nagoya University, Nagoya, Aichi, Japan

^3^Department of Medicine and Biological Science, Gunma University Graduate School of Medicine, Maebashi, Gunma, Japan

^4^Structural Glycobiology Team, RIKEN-Max Planck Joint Research Center for Systems Chemical Biology, RIKEN Global Research Cluster, Wako, Saitama, Japan

^5^Department of Respiratory Medicine and Allergology, Sapporo Medical University School of Medicine, Sapporo, Hokkaido, Japan

^6^Department of Cellular Pharmacology, Graduate School of Medicine, Hokkaido University, Sapporo, Hokkaido, Japan

^7^Department of Environmental Health Sciences and Molecular Toxicology, Tohoku University Graduate School of Medicine, Sendai, Japan

Supplementary Experimental Procedures

The detection of aldehyde adducts of SP-A by using an aldehyde reactive probe (ARP).

Aldehyde-protein adducts of SP-A were detected by labelling with *N*-(aminoxyacetyl)-*N’*-biotinylhydrazine (ARP, Dojindo, Kumamoto, Japan). An aliquot of lung lysate (500 μg protein) was incubated with biotinylated ARP (1 mM) at 37°C for 1 h. After removal of the unreacted ARP with a PD MidiTrap G-25 (GE), the collected protein fractions were incubated with NeutrAvidin agarose resin (Thermo Fisher scientific) overnight at 4°C. The beads were washed 6 times with PBS (1% NP40) and heated in SDS sample buffer. The eluted materials were separated by SDS-PAGE, then subjected to western blot analysis with the anti-SP-A antibody.

SP-A analysis by circular dichroism (CD) spectroscopy

The secondary structure of hSP-A was analysed by CD spectroscopy using a JASCO J-820 spectropolarimeter (Jasco, Tokyo, Japan: Center for Research and Education on Drug Discovery, Hokkaido University). hSP-A (25 μM) was incubated with acrolein (500 μM) at 37°C for 4 h. After removal of the excess unreacted acrolein with a PD SpinTrap G25, the samples were equilibrated in 20 mM sodium phosphate buffer (pH 7.4) and then diluted to 5 µM hSP-A in 20 mM sodium phosphate buffer. The far-UV CD spectra were acquired from 195 to 280 nm with 1 nm as the bandwidth, 2 s as the response time, 0.5 nm as the data pitch, and 10 nm/min as the scan speed.

Supplementary Figures


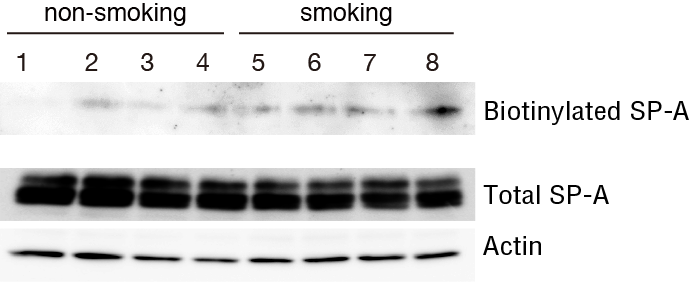


Supplementary Figure S1.

The expression of aldehyde adducts of SP-A in mouse lung after 1 week of exposure to CS.

Aldehyde adducts in lung tissue lysates were labelled by ARP and purified with NeutrAvidin agarose resin. The NeutrAvidin-purified ARP-labelled protein was analysed by western blotting with anti-SP-A antibody. A similar SP-A analysis in the lung tissue lysate (middle panel) is shown for comparison. Lanes 1–8 represent individual samples with n=4 mice per group. Uncropped blots are shown in Supplementary Fig. S6.


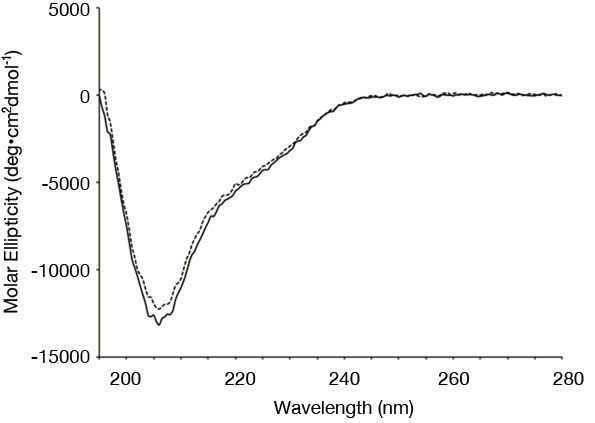


Supplementary Figure S2. The effects of acrolein modification on the secondary structure of SP-A.

Recombinant hSP-A was incubated with vehicle or acrolein (20:1 molar ratio of acrolein to hSP-A) at 37°C for 4 h. The structure of the hSP-A samples (5 μM protein in 20 mM sodium phosphate buffer) was then analysed by CD. Solid line: unmodified hSP-A; dotted line: acrolein-modified hSP-A.


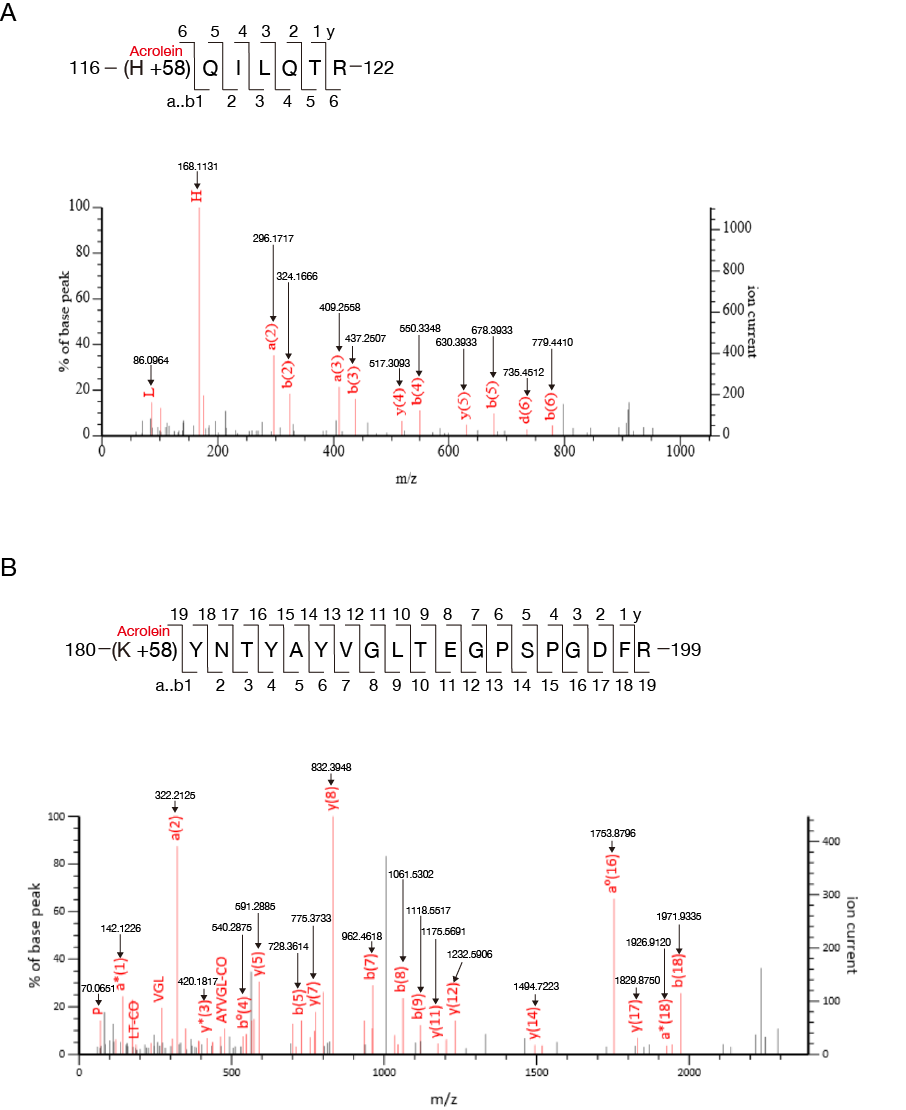


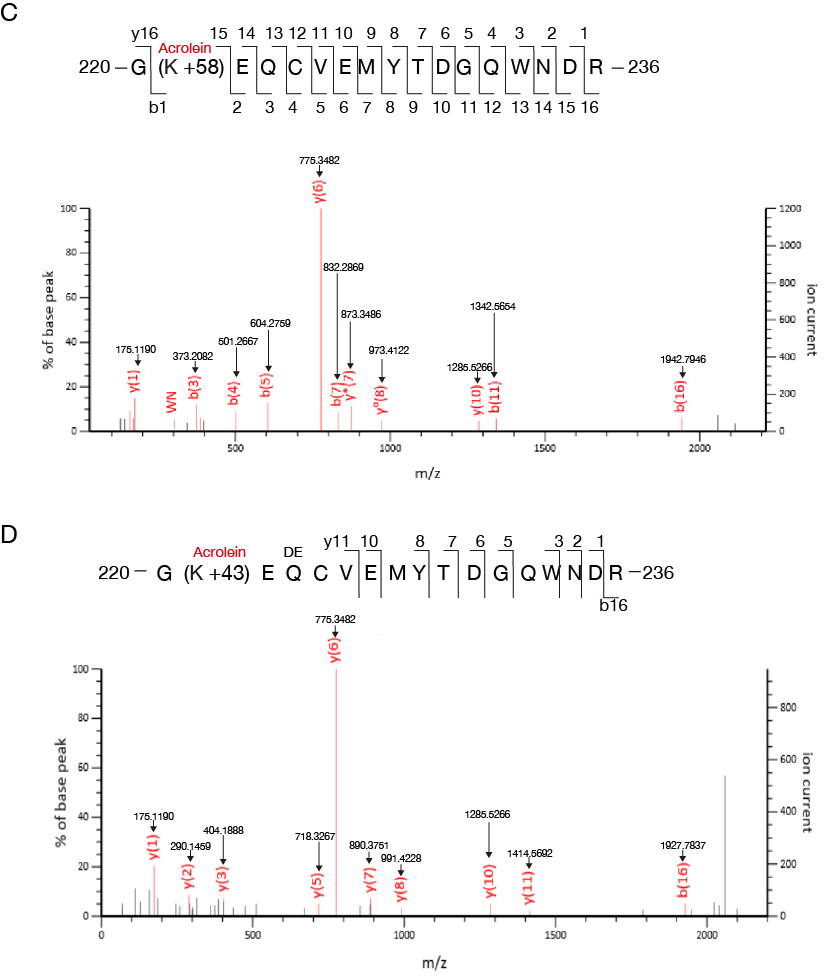


DE: deamitated

Supplementary Figure S3. Analysis of acrolein-modified hSP-A by nano-LC/MALDI-TOF MS/MS.

Recombinant hSP-A was incubated with vehicle or acrolein (20:1 molar ratio of acrolein to hSP-A) at 37°C for 4 h. After the NaBH_4_-treated samples were digested with trypsin, the peptides were subjected to nano-LC/MALDI-TOF MS/MS and analysed with the MASCOT Daemon software. There were four acrolein-modified residues in hSP-A. Three Michael addition-type acrolein-modified residues formed M+58 Da adducts; A: [H^116^+58QILQTR^122^], B: [K^180^+58YNTYAYVGLTEGPSPGDFR^190^], and C: [G^220^K^221^+58EQCVEMYTDGQWNDR^236^]. One Schiff-base reaction-type acrolein modified a residue to form M+43 Da adducts; D: [G^220^K^221^+43EQCVEMYTDGQWNDR^236^].


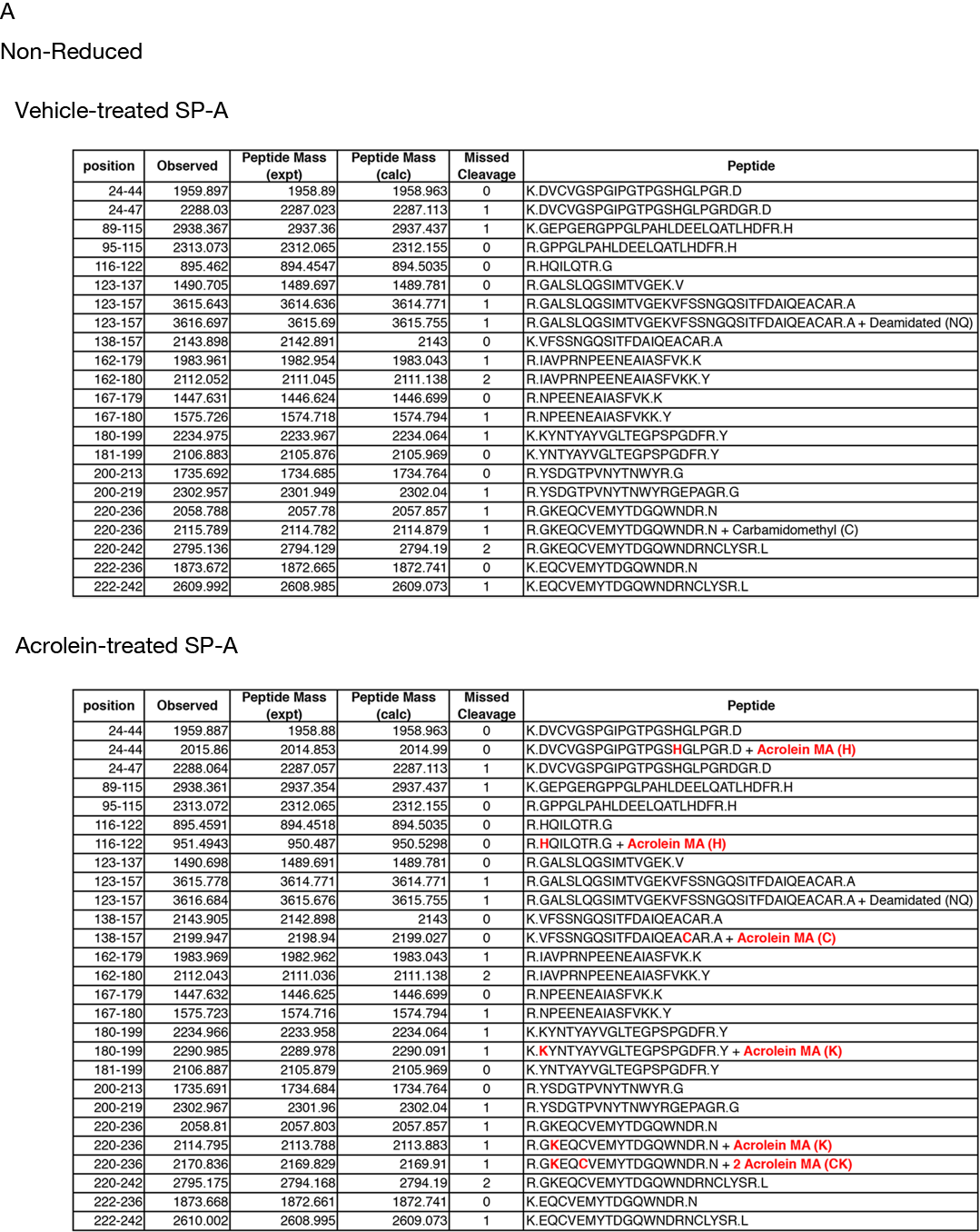


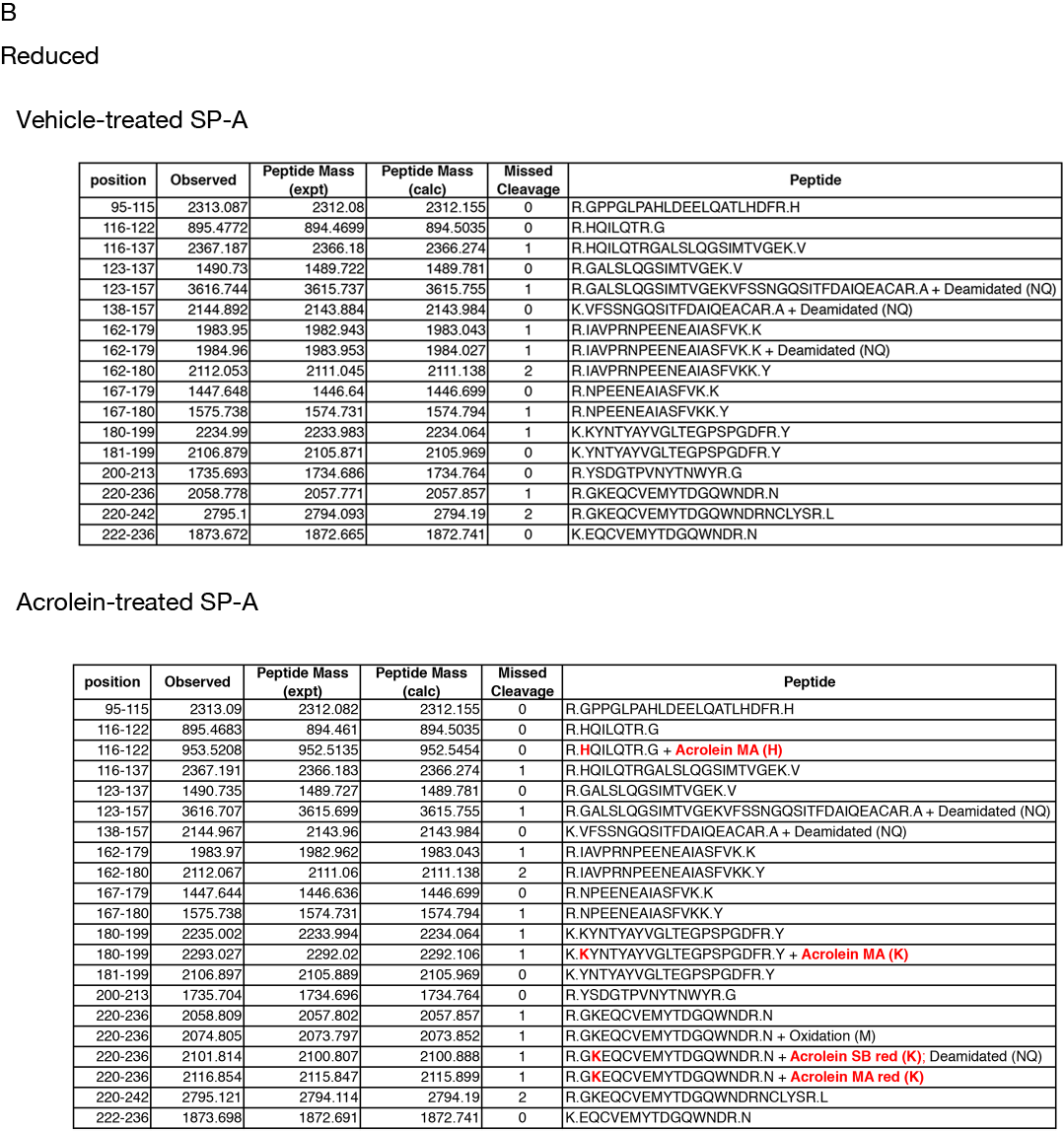


Peptide Mass (expt): experimental peptide mass

Peptide Mass (calc): peptide mass calculated for the indicated peptide sequence

MA: Michael adduct

MA red: Michael adduct reduced form
SB red: Schiff-base adduct reduced form

Supplementary Figure S4. The identification of acrolein-modified residues in SP-A.

(A) The MS/MS analysis of non-reduced unmodified (Upper) and acrolein-modified SP-A (Lower). (B) The MS/MS analysis of NaBH_4-_reduced unmodified (Upper) and acrolein-modified SP-A (Lower).


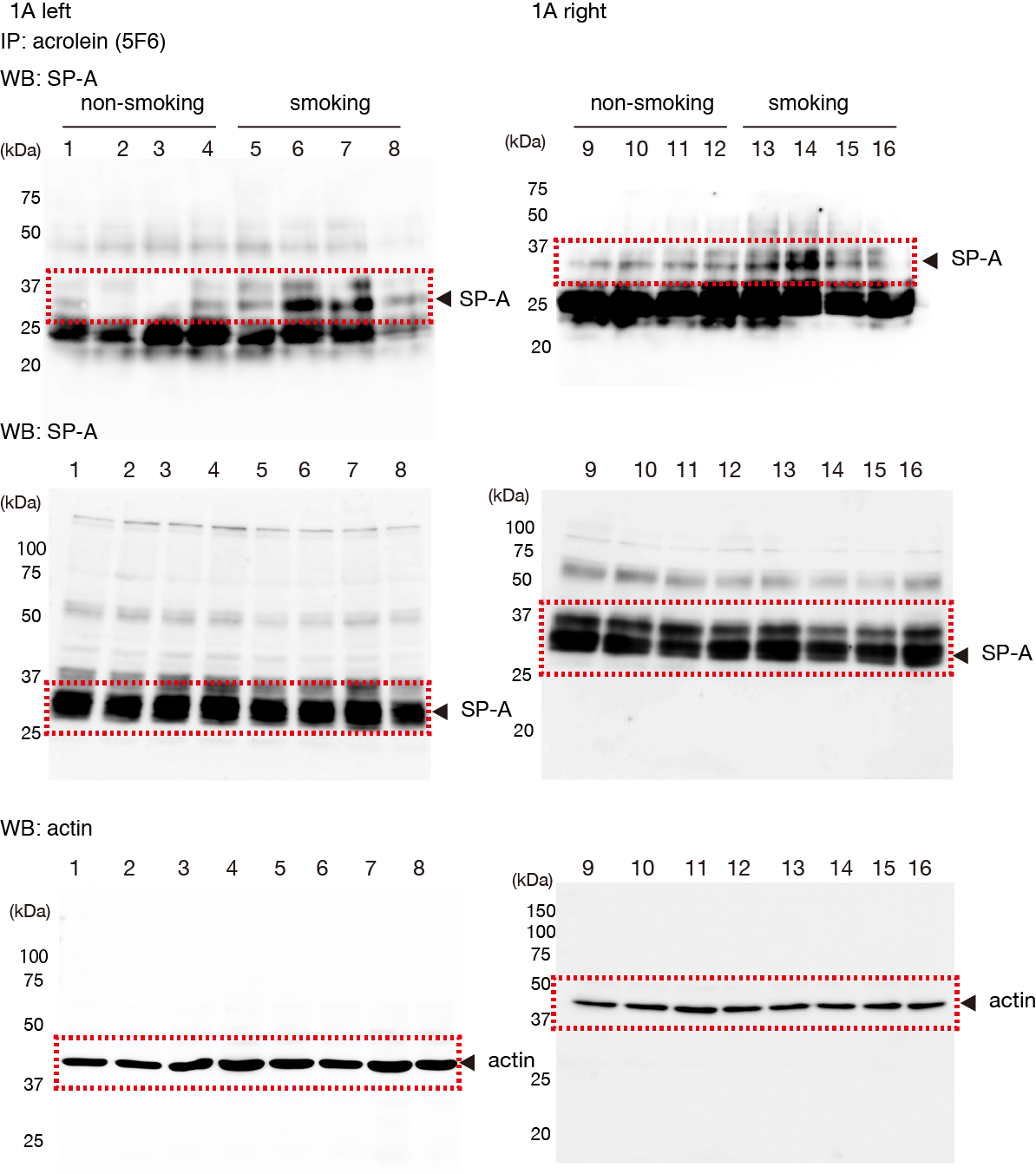


Supplementary Figure S5. Full uncropped blot images from Figure 1A.


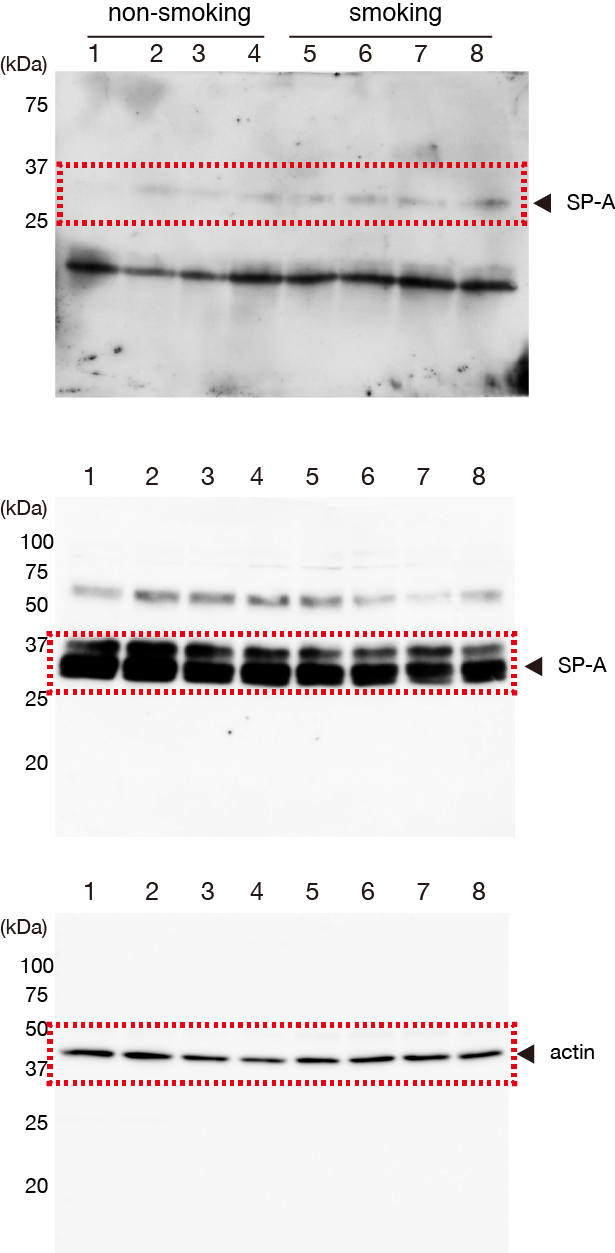


Supplementary Figure S6. Full uncropped blot images from Supplementary Figure S1.
